# Direct mechanisms of SARS-CoV-2-induced cardiomyocyte damage: an update

**DOI:** 10.1186/s12985-022-01833-y

**Published:** 2022-06-25

**Authors:** Yicheng Yang, Zhiyao Wei, Changming Xiong, Haiyan Qian

**Affiliations:** 1 Center for Coronary Heart Disease, Department of Cardiology, Fuwai Hospital, National Center for Cardiovascular Diseases of China, State Key Laboratory of Cardiovascular, Beijing, 100037 China; 2 Center for Pulmonary Vascular Diseases, Department of Cardiology, Fuwai Hospital, National Center for Cardiovascular Diseases of China, State Key Laboratory of Cardiovascular, Beijing, 100037 China; 3 Fuwai Hospital, National Center for Cardiovascular Diseases, Chinese Academy of Medical Sciences and Peking Union Medical College, 167 Beilishi Road, Xicheng District, Beijing, 100037 China

**Keywords:** SARS-CoV-2, COVID-19, Myocardial injury, Cardiomyocytes

## Abstract

Myocardial injury induced by severe acute respiratory syndrome coronavirus 2 (SARS-CoV-2) is reportedly related to disease severity and mortality, attracting attention to exploring relevant pathogenic mechanisms. Limited by insufficient evidence, myocardial injury caused by direct viral invasion of cardiomyocytes (CMs) is not fully understood. Based on recent studies, endosomal dependence can compensate for S protein priming to mediate SARS-CoV-2 infection of CMs, damage the contractile function of CMs, trigger electrical dysfunction, and tip the balance of the renin–angiotensin–aldosterone system to exert a myocardial injury effect. In this review, we shed light on the direct injury caused by SARS-CoV-2 to provide a comprehensive understanding of the cardiac manifestations of coronavirus disease 2019 (COVID-19).

## Background

The COVID-19 pandemic has resulted in more than 500 million confirmed cases worldwide as of April 2022. Myocardial injury induced by SARS-CoV-2 was reportedly attributable to disease severity and mortality. However, the relevant pathogenetic mechanisms of myocardial injury caused by direct viral invasion of cardiomyocytes have not been fully elucidated. To further understand the direct myocardial injury by SARS-CoV-2, we summarized the relative mechanisms in this review based on the latest knowledge.

## Introduction

Coronavirus disease 2019 (COVID-19), a contagious disease caused by the severe acute respiratory syndrome coronavirus 2 (SARS-CoV-2), has become a worldwide pandemic. The R0 of SARS-CoV-2 in the original strain was 2.5, whereas the number increased to approximately 7 in the delta variant (B.1.617.2). Another new variant of the virus, Omicron, first originated in southern Africa (South Africa, Botswana) and later spread to Britain and other countries. Omicron’s R0 could be as high as 10, indicating a more contagious phenotype than earlier virus variants. Omicron has become the current dominant variant worldwide, including in China [1, 2]. Although predominantly characterized by viral pneumonia, cardiac involvement could be prevalent during COVID-19 progression, which is reportedly associated with disease severity and mortality [3, 4].

SARS-CoV-2 invades cells via angiotensin-converting enzyme 2 (ACE2), a membrane protein that counterbalances the adverse effects of the renin–angiotensin–aldosterone system (RAAS) by converting angiotensin II (Ang II) to Ang-(1–7). It recognizes and binds to the ACE2 receptor via the spike (S) protein [5]. Wrapp et al. obtained the trimeric S protein structure by 3D reconstruction technology based on the genomic sequence of SARS-CoV-2 and observed that the binding affinity of the S protein to ACE2 was 10–20 fold higher compared to that of SARS-CoV, partly explaining its high contagiousness [6].

With the COVID-19 pandemic entering the third year, the role of SARS-CoV-2 in the heart and the cardiovascular system has gained attention [7–9]. Theoretically, cells expressing high ACE2 levels are more vulnerable to SARS-CoV-2 invasion and subsequent organ injury, including cardiomyocytes (CMs). According to clinical observations, increased myocardial biomarker levels unrelated to obstructive coronary artery disease, a condition generally diagnosed as myocardial injury, occurred in 7.2–40.9% of patients with COVID-19 [10]. This complication contributes to disease severity and mortality, with a hazard ratio ranging from 4.3 to 8.9 and an odds ratio from 6.6 to 26.9. Given the current convincing evidence that myocardial injury intensifies the severity of COVID-19, cardiac management should be a priority for physicians. In addition, the long-term effects of COVID-19 on cardiovascular health are still a major global concern [4]. We have previously proposed several pathogenic mechanisms explaining the association of COVID-19 with myocardial injury, including SARS-CoV-2 host cell invasion, myocardial oxygen supply/demand imbalance, abnormal coagulation, and excessive immune reaction [11]. However, limited by insufficient evidence, myocardial injury caused by direct viral invasion of CMs was not fully understood earlier. Given the rapid development of this field, in this review, we shed light on the updated mechanisms of CM impairment associated with direct SARS-CoV-2 invasion to provide a more comprehensive understanding of the cardiac manifestations in COVID-19.

### Direct and indirect SARS-CoV-2 entry into CMs

Pathology reports from several studies have identified SARS-CoV-2 viral proteins and genetic material in CMs of patients with COVID-19 [12–15], even in those with no clinical signs of cardiac involvement [16, 17]. In addition, in vitro studies with human induced pluripotent stem cells (hiPS) and isolated adult CMs and in vivo experiments using animal models have confirmed that CMs are permissive for SARS-CoV-2 infection [14, 15, 18]. Bojkova et al. successfully infected hiPS-derived CMs with SARS-CoV-2 and demonstrated the presence of intracellular double-strand viral RNA and viral spike glycoprotein protein expression [14]. Increasing concentrations of viral RNA were detected in the supernatants of infected CMs, and SARS-CoV-2 infection ability was confirmed in 3D cardiosphere tissue. Overall, these data support the hypothesis that COVID-19-associated myocardial injury can result from the direct infection of CMs and the cardiotoxic effect of SARS-CoV-2 and is not merely a secondary effect derived from hypoxia and systemic inflammation.

A single-cell sequencing study has revealed that the expression of viral ACE2 receptors is noticeable in CMs from normal hearts and has suggested that ACE2 expression in the adult human heart is higher than that in the lung [19]. Furthermore, ACE2 expression was significantly elevated in CMs of patients with heart disease (e.g., dilated cardiomyopathy, hypertrophic cardiomyopathy, non-COVID-19 myocarditis, aortic stenosis, and heart failure) compared to healthy controls [20–22]. These findings are consistent with the clinical observations and epidemiologic reports suggesting that patients with underlying cardiovascular conditions are more vulnerable and prone to myocardial injury [11, 23].

Type II transmembrane serine protease (TMPRSS2) is the protein involved in the cleavage of many SARS-CoV-2 variants. TMPRSS2 cleaves the S protein into the S1 and S2 subunits, exposing the receptor-binding domain (RBD) of the S1 subunit to facilitate recognition and binding to the ACE2 receptor. Notably, SARS-CoV-2 entry into CMs is mediated by an endosomal-dependent mechanism that does not require TMPRSS2, unlike lung-derived cell lines where TMPRSS2 mediates viral entry [13, 14, 19]. Contrary to TMPRSS2, multiple endosomal proteases, including cathepsins and calpains, are highly expressed at a level similar to that in the lungs in human pluripotent stem cell-derived CMs (hPSC-CMs) and CMs from human autopsy specimens, [14, 19]. Bailey et al. inhibited TMPRSS2 and found no effect on hPSC-CMs infection, whereas an endosomal cysteine protease inhibitor abolished SARS-CoV-2 infection [13]. These data suggest that the endosomal-dependent proteases compensate for S protein priming to mediate SARS-CoV-2 infection in CMs. However, Omicron, a recent variant of SARS-CoV-2, only requires ACE2 to invade the cell without the help of TMPRSS2. This variant is encapsulated in endosome bubble, which drifts into the cells and breaks out [24]. Therefore, cells without or with less TMPRSS2 expression are easily infected by Omicron, resulting in relevant damage, and theoretically, CMs are more vulnerable to virus invasion leading to myocardial damage. In conclusion, the specific effects of Omicron on cardiac injury need to be further explored.

In addition to direct attacks, SARS-CoV-2 may also infect CMs indirectly. Kwon et al. isolated extracellular vesicles from lung epithelial cells that overexpressed SARS-CoV-2 genes and found that hiPSC-CMs were receptive to these viral RNA-harboring extracellular vesicles [25]. Viral genes were detected in hiPSC-CMs, along with inflammatory activation.

### Pathogenic mechanisms of direct SARS-CoV-2 infection

Recent reports have identified SARS-CoV-2 particles or components within CMs from autopsy and endomyocardial biopsy samples utilizing RNA sequencing, RNAscope, immunohistochemistry, and transmission electron microscopy analysis Direct viral infection leads to adverse effects on CMs in several aspects, such as cell morphology, electrophysiology, subcellular structures, and cell death [15, 16, 26–29].

### Direct infection can damage the contractile function of CMs

Direct injury to the heart tissue by SARS-CoV-2 could be an underlying cause of heart disease in COVID-19 [30]. Myocardial contractile dysfunction is one of the prominent manifestations of COVID-19-related cardiac complications. The significant reduction in cardiac contractility and subsequent myocardial dysfunction induced by SARS-CoV-2 has received widespread attention. The European Society of Cardiology has published a consensus to elucidate the current knowledge of cardiovascular damage induced by SARS-CoV-2 for alteration in the clinic [31].

The pathogenic mechanisms of SARS-CoV-2 are commonly considered to be multifaceted. One of these mechanisms is based on data indicating that virus infection may directly destroy sarcomeres in CMs. Sarcomeric disruptions and myofibril loss in CMs have been observed occasionally in vivo and in vitro, and both presented similar signatures [14, 16, 32]. Pérez-Bermejo et al. observed that the number of myofibrillar fragments produced by cleaving sarcomeres into individual sarcomeric units increased gradually in the cytoplasm of hiPSC-CMs cytoplasm as the virus exposure time increased, suggesting that the invasion of SARS-CoV-2 exacerbated this non-specific cytopathy. Furthermore, high-content imaging analysis of infected hiPSCs-CMs showed that these fragments and intact sarcomeres possessed identical structures, except for the absence of a dark A-band in the myofibrillar fragments, suggesting the cleavage of thick filaments. Notably, they observed the LKGG↓K sequence in myosin heavy chain protein family members. This sequence is similar to those in viral polyproteins that can be cleaved by the SARS-CoV-2 papain-like protease. This result indicates that the virus-derived proteases, indicating besides processing their target sequences, may possess the capacity to cleave several intracellular proteins and alter the cellular structural integrity [29].

Another pathogenic mechanism of SARS-CoV-2 is related to the fact that the virus damages contractility by disrupting gene expression profiles, particularly of those genes directly involved in sarcomere function. Significant downregulation of genes encoding sarcomeric structural proteins, myosin light chains, and linker of nucleoskeleton and cytoskeleton complex (a subset of proteins that are important for anchoring the nucleus to the actin cytoskeleton) in infected hiPSCs-CMs. Furthermore, study revealed alterations in the expression of genes related to energy production leading to a shift in the energy production mechanism from mitochondrial oxidative phosphorylation to glycolytic metabolism in hiPSC-CMs and hPSC-CMs [18]. The resulting energy reduction can adversely affect contraction at the cellular and tissue levels. Although oxidative stress and inflammation may directly alter gene expression profiles in infected CMs, and other effectors as the non-structure protein 1 (Nsp1) may also play a role. Nsp1 binds to the 40S ribosomal subunit in a manner that its C-terminal domain directly blocks the entry channel of messenger RNA in the ribosome, perturbing the translation activities of host cells and shutting down the translation of host messenger RNA [33]. Collectively, these factors may result in the breakdown of the contractile machinery and the appearance of impaired myocardial contractility.

To evaluate the influence of SARS-CoV-2 infection on cardiac tissue in vivo, Siddiq et al. measured the damage severity of hiPSC-CMs treated with interleukins (ILs) or vehicle in the presence or absence of SARS-CoV-2 infection. ILs treatment alone did not increase the release of troponin-I (a marker for damaged myofibrils) but augmented the cytopathic effect of infection in hiPSC-CMs [34]. These results further emphasize the prominent role of direct SARS-CoV-2 infection in CMs damage and indicate the facilitative role of ILs in vivo. Moreover, researchers have utilized three-dimensional engineered heart tissues (3D-EHTs) to further evaluate the contractile properties of hPSC-CMs at the tissue level and observed that 3D-EHTs infected with SARS-CoV-2 presented weakening contractile forces compared to mock controls [13, 18]. These results shed light on the correlations between the contractile dysfunction at the cellular level induced by direct infection and the prevalent myocardial injury in patients with COVID-19 at the organ-tissue level. However, further investigations are warranted to elucidate the underlying mechanisms thoroughly.

### Direct infection can trigger electrical dysfunction of CMs

Patients with COVID-19 are more prone to suffering heart damage or irregular heartbeats, indicating the presence of CMs dysfunction induced by SARS-CoV-2 [35]. In addition to mechanical dysfunction, SARS-CoV-2 infected CMs also have impaired electrical functions. Marchiano et al. discovered that SARS-CoV-2 infection resulted in the abnormal generation and propagation of electrical signals in hPSC-CMs, hiPSC-CMs, and human embryonic stem cell-derived CMs (hESC-CMs), which show reduced beating rate, lower depolarization spike amplitude, and decreased electrical conduction velocity. They also observed a time-dependent increase in the field potential duration (FPD) in H7 hESC-CMs in both spontaneously beating and electrically-paced cultures [18]. Anomalous electrical signals manifest even in the absence of extensive cell death, suggesting that there may be some virus-derived components or subsequently generated substrates that disrupt cellular homeostasis, thus causing electrical dysfunction at the cellular and even at the tissue-organ level. Interestingly, a recent study proposed that SARS-CoV-2 infection-induced CM structural alterations lead to electrophysiological abnormalities. Some researchers have identified syncytia formation in SARS-CoV-2 infected cells and tissue, which is relatively rare but not absent compared to other cytopathies [36, 37]. Coronaviruses commonly induce cell–cell fusion because of the fusogenic property of the S protein and its capability to trigger virus-cell membrane fusion [38]. Schneider et al. identified intracellular junctions between CMs formed by highly-concentrated sarcolemmal t-tubule viral S protein in myocardial specimens from a young woman who died of sudden cardiac death and was found to be COVID-19-positive during the postmortem. The S protein likely tends to promote the formation of junctions between adjacent CMs instead of syncytia due to cytoskeletal constraints and viral infectivity [37, 38]. These cell-to-cell conduits are prone to short-circuit electrically excitable myocardium, and give rise to electrophysiological abnormalities. Moreover, marked pathological Ca^2+^ flux, sparks, and tsunami-like waves have been observed in hiPSC-CMs built multinucleated cardiomyotubes, which present significantly prolonged action potential duration, elevated membrane capacitance, delayed afterdepolarizations, and erratic beating frequencies frequency compared to mock controls. Altogether, these studies provide a novel perspective on the pathogenic mechanism of COVID-19-related arrhythmias and reiterate the detrimental effects of direct SARS-CoV-2 infection in CMs.

### Direct infection tips the balance of RAAS towards cardiac damage

**Fig. 1:**
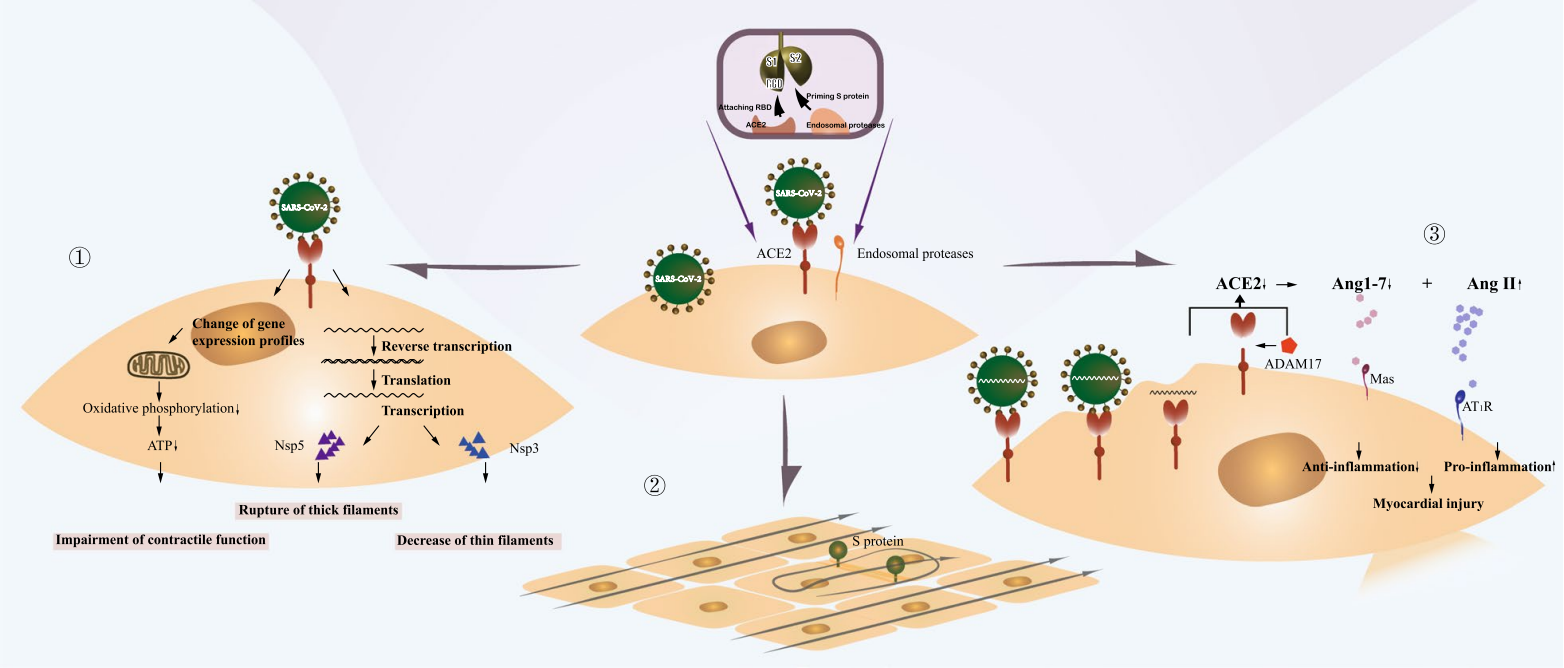
Mechanisms of direct myocardial injury induced by SARS-CoV-2. SARS-CoV-2 can enter into CMs through an endosomal-dependent mechanism. Cleaving the S protein into the S1 and S2 subunits exposes the RBD of the S1 subunit, allowing the virus to recognize and bind to the ACE2 receptor. SARS-CoV-2 invasion may cause direct myocardial injury through three mechanisms. (1) Nsp3 and Nsp5 production lead to the decrease of thin filaments and rupture of thick filaments, respectively. In addition, SARS-CoV-2 infection impairs the contractile function of CMs by altering gene expression profiles and reducing the mitochondrial oxidative phosphorylation levels. (2) The S protein promotes the formation of junctions between adjacent CMs and causes electrophysiological abnormalities. (3) Endocytosis and cleavage of ACE2 result in high AngII and low Ang (1–7) levels, leading to a decrease in anti-inflammatory processes and cardiomyocyte injury. CMs: cardiomyocytes; ACE2: angiotensin-converting enzyme 2; RBD: receptor-binding domain; Nsp: non-structure protein; ATP: adenosine triphosphate; Ang: angiotensin; ADAM17: A disintegrin and Metalloproteinase-17; AT1R: angiotensin II type-1 receptor

Another widely recognized alteration induced by SARS-CoV-2 infection is the loss of ACE2 and the subsequent events in CMs. ACE2 is a homolog of ACE and negatively regulates RAAS by degrading AngII to Ang (1–7), whereas ACE metabolizes AngI to AngII. Reduction of ACE2 on the CM membrane is induced by suppressed gene expression, enhanced endocytosis, and cleavage by A disintegrin and metalloproteinase-17 (ADAM-17), a transmembrane protease evoked by the sudden-increased in ILs after infection. Decreased ACE2 undermines its counterbalance effect on the AngII/Ang II type-1 receptor pathway and results in the accumulation of AngII and attenuation of the Ang (1–7)/Mas receptor pathway. Excessive AngII induces overt CM autophagy and apoptosis, which is speculated to play a critical role in developing decompensated hypertensive heart disease [39, 40]. Contrary to AngII, Ang (1–7) and Mas are considered cardioprotective factors. Liu et al. observed that Ang (1–7) prevents chronic atrial ionic remodeling by suppressing the decrease of transient outward potassium current (Ito) and L-type Ca^2+^ current (ICaL), as well as the reduction of Kv4.3 subunit gene expression [41]. In addition, Ang (1–7) is reportedly effective in counteracting the increase in isoproterenol-induced beating rate in CMs, reflecting its potential to attenuate cardiac reactivity in response to acute emotional stress [42]. Dias-Peixoto et al. discovered that CMs from Mas-deficient mice are characterized by reduced peak and slower [Ca^2+^] transients, suggesting that depression of the Mas receptor pathway might disturb the Ca^2+^-handling capacity of CMs [43]. Thus, during SARS-CoV-2 infection, the diminished cardioprotective effects and hyperactive harmful processes can synergize and lead to significant CMs damage in various ways. The different mechanisms of direct myocardial injury induced by SARS-CoV-2 are shown in Fig. 1.

## Conclusions

The COVID-19 pandemic has resulted in health concerns worldwide. Myocardial damage induced by SARS-CoV-2 has received unprecedented attention because of its direct relation to disease severity and increased mortality in patients with COVID-19. SARS-CoV-2 can invade CMs by recognizing and binding to ACE2 receptors on the cardiomyocyte membrane and endosomal-dependent mechanisms. To improve clinical management and patient prognosis, the relevant mechanisms of myocardial injury caused by SARS-CoV-2 must be explored. We have, thus, reviewed recent studies that shed light on the direct mechanisms of SARS-CoV-2 injury on the myocardium and conclude that viral invasion damages the contractile function of CMs, triggers electrical dysfunction, and tips the RAAS balance to cause myocardial injury. However, further investigations are warranted to elucidate the underlying mechanisms for better awareness and treatment.

## Data Availability

Not applicable.

## Abbreviations

SARS-CoV-2: Severe acute respiratory syndrome coronavirus 2
COVID-19: Coronavirus disease 2019
CMs: Cardiomyocytes
hiPS: Human induced pluripotent stem cells
TMPRSS2: Type II transmembrane serine protease
RBD: Receptor-binding domain
hPSC-CMs: Human pluripotent stem cell-derived CMs
ACE2: Angiotensin-converting enzyme 2
RAAS: Renin–angiotensin–aldosterone system
Nsp1: Non-structure protein 1
ILs: Interleukins
hESC-CMs: Human embryonic stem cell-derived CMs
FPD: Field potential duration
ADAM-17: A disintegrin and metalloproteinase-17
Ito: Transient outward potassium current
ICaL: L-type Ca^2 +^ current
